# Outward- and inward-facing structures of a putative bacterial transition-metal transporter with homology to ferroportin

**DOI:** 10.1038/ncomms9545

**Published:** 2015-10-13

**Authors:** Reiya Taniguchi, Hideaki E. Kato, Josep Font, Chandrika N. Deshpande, Miki Wada, Koichi Ito, Ryuichiro Ishitani, Mika Jormakka, Osamu Nureki

**Affiliations:** 1 Department of Biological Sciences, Graduate School of Science, The University of Tokyo, 2-11-16 Yayoi, Bunkyo-ku, Tokyo 113-0032, Japan; 2 Global Research Cluster, RIKEN, 2-1 Hirosawa, Wako-shi, Saitama 351-0198, Japan; 3 Structural Biology Program, Centenary Institute, Locked Bag 6, Sydney, New South Wales 2042, Australia; 4 Faculty of Medicine, Central Clinical School, University of Sydney, Sydney, New South Wales 2006, Australia; 5 Technical office, The Institute of Medical Science, The University of Tokyo, 4-6-1 Shirokanedai, Minato-ku, Tokyo 108-8639, Japan; 6 Department of Medical Genome Sciences, Graduate School of Frontier Sciences, The University of Tokyo, 5-1-5 Kashiwanoha, Kashiwa-shi, Chiba 277-8562, Japan

## Abstract

In vertebrates, the iron exporter ferroportin releases Fe^2+^ from cells into plasma, thereby maintaining iron homeostasis. The transport activity of ferroportin is suppressed by the peptide hormone hepcidin, which exhibits upregulated expression in chronic inflammation, causing iron-restrictive anaemia. However, due to the lack of structural information about ferroportin, the mechanisms of its iron transport and hepcidin-mediated regulation remain largely elusive. Here we report the crystal structures of a putative bacterial homologue of ferroportin, BbFPN, in both the outward- and inward-facing states. Despite undetectable sequence similarity, BbFPN adopts the major facilitator superfamily fold. A comparison of the two structures reveals that BbFPN undergoes an intra-domain conformational rearrangement during the transport cycle. We identify a substrate metal-binding site, based on structural and mutational analyses. Furthermore, the BbFPN structures suggest that a predicted hepcidin-binding site of ferroportin is located within its central cavity. Thus, BbFPN may be a valuable structural model for iron homeostasis regulation by ferroportin.

## Abstract (web summary)

Iron export from vertebrate cells is mediated by ferroportin, which is suppressed by the peptide hormone hepcidin. Taniguchi *et al*. present crystal structures of a putative bacterial ferroportin homologue in both outward- and inward-facing states, providing insight into its transport mechanism.

## Graphical abstract

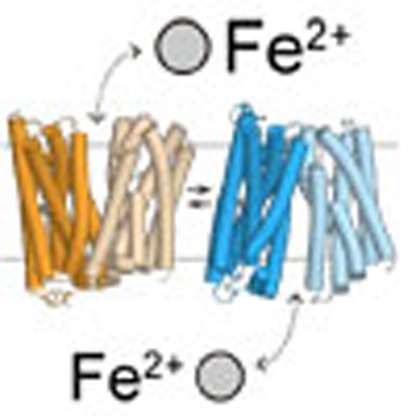

Iron is an essential nutrient for all eukaryotic and most prokaryotic organisms. In humans, iron plays a critical role in a large array of biological processes, including oxygen transport in blood and enzymatic reduction/oxidation reactions. However, iron is also highly toxic; its elevated levels cause oxidative stress, which finally leads to cellular dysfunctions, including cancer, neurodegenerative diseases and cell death. Therefore, iron absorption, distribution and recycling in the body are strictly regulated to properly maintain the systemic iron homeostasis.

The cellular iron exporter ferroportin (SLC40A1, FPN)^1^,^2^,^3^ plays a critical role in mammalian iron homeostasis, and is the only known iron exporter to date. FPN exports iron in the ferrous (Fe^2+^) form, and is essential for the delivery of dietary absorbed, stored and recycled iron into plasma from duodenal enterocytes, hepatocytes and macrophages^2^,^4^,^5^, respectively. FPN is also indispensable for the transport of iron into the placenta during pregnancy, and the complete inactivation of FPN is embryonically lethal^6^. Mutations in the FPN gene that affect the FPN transport activity cause iron overload disease, known as type 4 hereditary hemochromatosis or ferroportin disease^7^,^8^. The level of active FPN in the plasma membrane is negatively regulated by the small peptide hormone hepcidin^9^,^10^, which is released from hepatocytes when systemic iron levels are sufficient^11^. Hepcidin binding to FPN induces the subsequent ubiquitination-dependent internalization and lysosomal degradation of the hepcidin–FPN complex, thereby downregulating the cellular iron export activity^9^,^12^,^13^. During chronic inflammation, infection or malignancies, the production of hepcidin is upregulated due to elevated serum levels of inflammatory cytokines, particularly interleukin-6, leading to drastic reductions in the active FPN and plasma iron levels^14^,^15^. This leads to iron-restrictive anaemia, or anaemia of chronic inflammation, which causes increased recovery times and mortality rates in hospitalized patients^16^. In contrast, unrestrained FPN activity due to reduced hepcidin levels leads to high serum-iron levels, causing iron overload diseases such as β-thalassaemia^15^,^17^.

Given the central roles that FPN and hepcidin play in cellular iron homeostasis and iron disorder pathogenesis, the detailed mechanisms of iron transport and regulation have profound biomedical significance. However, the fundamental aspects regarding the three-dimensional structure and iron transport mechanism of FPN are largely unknown. A previous biochemical analysis suggested that FPN has 12 transmembrane (TM) helices^18^, and several structural models of FPN have been constructed based on the crystal structures of major facilitator superfamily (MFS) transporters, which also possess 12 TM segments^19^,^20^,^21^,^22^. These models and mutational analyses suggested that the hepcidin-binding site of FPN exists on an extracellular loop. However, due to the low sequence homology between FPN and MFS transporters with known structures, no reliable consensus model has been obtained to elucidate the detailed structural basis for iron transport and hepcidin-mediated downregulation.

To gain insight into the structure and function of FPN, we determined the crystal structures of its putative bacterial homologue in two distinct states. These structures indicate that the FPN family shares a common TM topology with MFS transporters, thus illustrating the first MFS transporter that mediates cation transport. A comparison of the structures in the two distinct states reveals the conformational rearrangements involving TM bending in the transport cycle. Our results also illuminate a putative mechanism of hepcidin-mediated FPN inhibition, thus suggesting new approaches for the development of drugs to treat iron-related disorders.

## Results

### Functional characterization of a putative FPN homologue

**Figure 1:**
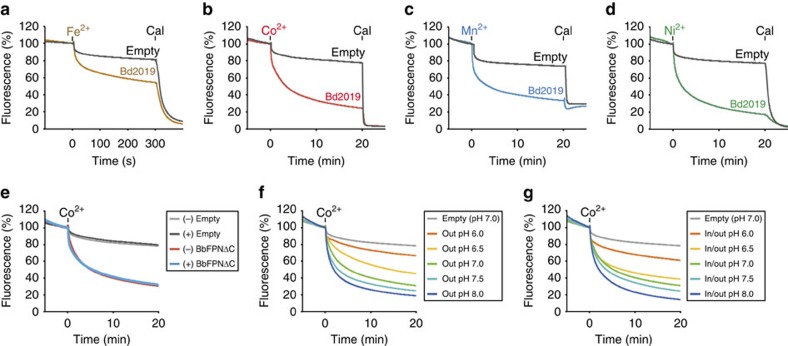
Functional characterization of BbFPN. (**a**–**d**) BbFPN-mediated transport of (**a**) Fe^2+^, (**b**) Co^2+^, (**c**) Mn^2+^ and (**d**) Ni^2+^. Time-dependent quenching of calcein fluorescence inside the liposomes is shown. Addition of transition-metal cation and the ionophore calcimycin (Cal) is shown above the graphs. (**e**–**g**) The transport activity of BbFPN measured with (**e**) the presence (+) or absence (−) of the ionophore gramicidin, (**f**) the pH gradient across the membrane, or (**g**) the different pH of the buffer solution. In **e**–**g** the pH of the buffer solution is pH 7.0 unless otherwise stated in the boxes. The control data of empty liposomes were measured at pH 7.0. All measurements were repeated three times, and representative data are shown.

We identified the hypothetical iron transporter Bd2019 from the recently sequenced bacterium *Bdellovibrio bacteriovorus*, with 24% sequence identity and 40% similarity with human FPN (hFPN) (Supplementary Fig. 1). The GFP-fused Bd2019 exhibited a monodisperse peak in the fluorescent size exclusion chromatography analysis^23^, indicating its suitability for crystallization (Supplementary Fig. 2a). Bd2019 is the only bacterial membrane protein showing sequence homology to the FPNs, and it has not been functionally characterized. To determine the transport substrates of Bd2019, we reconstituted the purified protein into liposomes and analysed the cation transport activity *in vitro*. Using the divalent cation indicator calcein^24^, we detected the Bd2019-mediated transport of Fe^2+^, Co^2+^, Mn^2+^ and Ni^2+^ into proteoliposomes (Fig. 1a–d). These results clearly indicated that Bd2019 is a divalent cation transporter selective to transition metals, including Fe^2+^. Notably, recent studies showed that hFPN also has Co^2+^ and Mn^2+^ transport activities^25^,^26^, further supporting the functional similarity between Bd2019 and FPNs. Taken together, we considered that Bd2019 is a putative homologue of FPN, and hereafter refer to the protein as BbFPN. Due to the difficulty of handling Fe^2+^ under aerobic conditions, we used Co^2+^ instead for the further functional analyses.

The addition of the non-selective monovalent cation ionophore gramicidin into the assay system did not affect the BbFPN-mediated cation transport (Fig. 1e), suggesting that the translocation of other monovalent cations, such as protons and Na^+^ ions, is not strictly required for the transport activity of BbFPN. Supporting this notion, the concentration difference of Na^+^ between the inner and outer solutions of the liposomes had no influence on the transport activity (Supplementary Fig. 2b). When the pH of the outer solution was changed, a pH-dependent difference in the transport activity was observed (Fig. 1f), with higher activities at higher pH values. However, a similar pH-dependent difference was also observed when the pH values of both the inner and outer solutions were changed (Fig. 1g), showing that the difference in the transport activity is actually due to the pH sensitivity of the BbFPN activity. These results indicate that the transport of transition-metal cations is unlikely to be driven by proton or Na^+^ gradients. Although we cannot fully exclude the possibility that other ions participate in the transport process, our experimental data are consistent with the notion that BbFPN functions in a uniporter-like manner, in which divalent metal cations are transported along their concentration gradient.

### Structure determination of BbFPN

For the structural analysis of BbFPN, we first purified the full-length protein and crystallized it using the lipidic cubic phase crystallization method^27^,^28^, which yielded crystals diffracting to a resolution of 7 Å. To improve the quality of the crystals, we removed eight C-terminal residues of BbFPN, which were predicted to be disordered. The liposome assay revealed that this truncated construct (BbFPNΔC) retains similar cation transport activity to that of the full-length BbFPN (Supplementary Fig. 2c). This C-terminal truncation improved both the size and diffraction properties of the crystals, and we finally obtained crystal form I, which diffracted to a resolution above 3 Å. The phase information was derived from a mercury-derivatized crystal, using the single isomorphous replacement with anomalous scattering method, and the structure of crystal form I was determined at 2.2 Å resolution (Table 1, see Methods). Further crystallization screening enabled us to obtain another crystal form (form II), and its structure was determined at 3.3 Å resolution by molecular replacement, using the crystal form I structure as a search model (Table 1).

**Figure 2:**
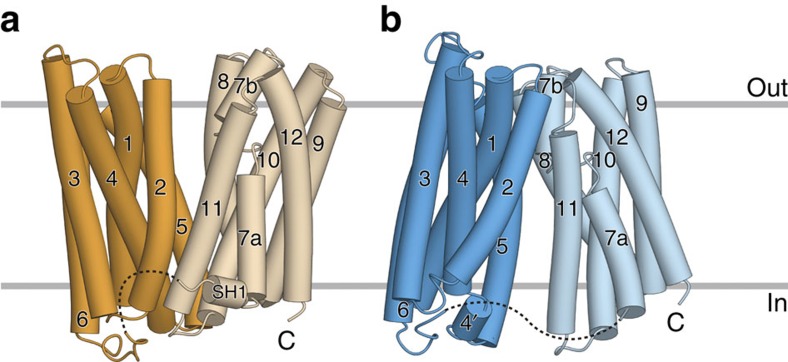
Overall structures of BbFPN. Overall structures of BbFPN in (**a**) the outward-facing state and (**b**) the inward-facing state. In **a** the N lobe and C lobe of the outward-facing structure are coloured orange and pale orange, respectively. In **b** the N lobe and C lobe of the inward-facing structure are coloured blue and pale blue, respectively.

**Figure 3:**
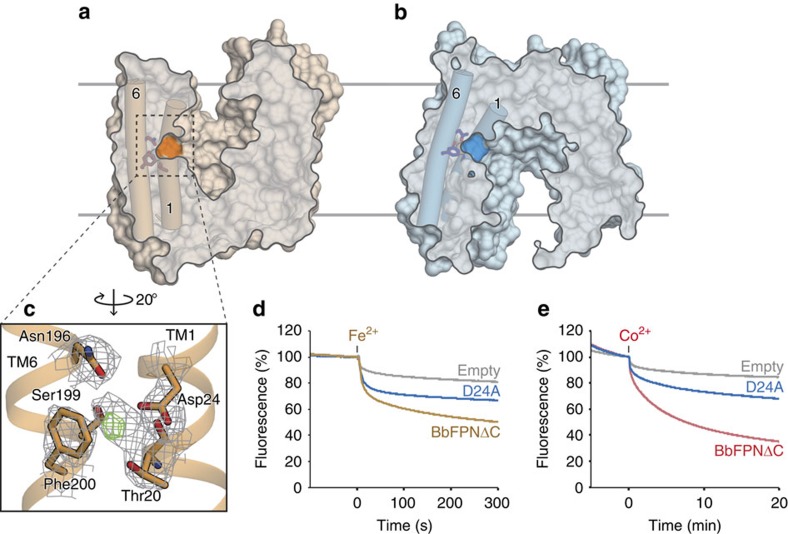
Metal-binding site. (**a**,**b**) Cut away surface representations of (**a**) the outward-facing and (**b**) the inward-facing structures of BbFPN, viewed from the same orientation as in Fig. 2, visualizing the central cavity and the metal-binding site. The cross sections are shown semitransparently to visualize TM1 and TM6, which are shown as cylinders. The positions of the metal-binding residues are highlighted on the molecular surface, and also shown as stick models. (**c**) Close-up view of the Fe^2+^-binding site. The anomalous difference density of Fe^2+^ (contoured at 4*σ*) is shown as green mesh, and the refined 2*F*o−*F*c density (contoured at 1.5*σ*) is shown as grey mesh. The Fe^2+^ coordinating residues are shown as stick models. (**d**,**e**) The (**d**) Fe^2+^ and (**e**) Co^2+^ transport activities of the Asp24Ala mutant, measured using calcein fluorescence. The blue trace indicates the results from the Asp24Ala mutant. The liposome measurements were repeated three times, and representative data are shown.

Consistent with the predicted topology of hFPN^18^, the crystal structures of BbFPN contain 12 TM helices. The overall structure of crystal form I exhibited the typical MFS fold^29^,^30^. Similar to the previously reported MFS structures, the 12 TM helices are divided into two halves, with TM1 to TM6 forming the N lobe (residues 7–213) and TM7 to TM12 forming the C lobe (residues 242–426) (Fig. 2a). These two lobes are related by twofold pseudosymmetry, and are connected by a long cytosolic loop (214–241) between TM6 and TM7, in which residues 225–232 are disordered. Residues 235–240 form a short helix (SH1) running parallel to the membrane plane, near the intracellular end of TM7. A central cavity exists between the N and C lobes, and is open towards the extracellular side and not accessible from the intracellular side (Fig. 2a; Fig. 3a). Accordingly, the form I structure superimposes well with the outward-facing structures of MFS transporters (for example, FucP^31^ with a root-mean squared deviation (r.m.s.d.) of 3.12 Å over 311 Cα atoms; Supplementary Fig. 3a). Thus, we refer to the form I structure as the outward-facing state. Despite the shared overall fold with MFS, a distinct structural feature exists in TM7: an unwound region at its centre separates the helix into a longer TM7a and a shorter TM7b (Fig. 2a; Supplementary Fig. 4a). This structural feature is not observed in other MFS proteins with known structures.

The form II structure also comprises the pseudosymmetry-related N and C lobes, and their structures are respectively similar to those of the outward-facing structure, including the unwound TM7 (Fig. 2b; Supplementary Fig. 4b). The loop connecting the N and C lobes is mostly disordered, and the density between residues 223 and 239 was not observed. Strikingly, the orientations of the N and C lobes are completely different from those of the outward-facing structure: the cavity between the two lobes is open towards the intracellular side and not accessible from the extracellular side (Fig. 2b; Fig. 3b). Correspondingly, the form II structure can be superimposed well onto the inward-facing structures of MFS transporters (for example, GkPOT^32^ with an r.m.s.d. of 3.72 Å over 328 Cα atoms; Supplementary Fig. 3b). Thus, we refer to the form II structure as the inward-facing structure.

### Transition-metal ion binding site

**Figure 4:**
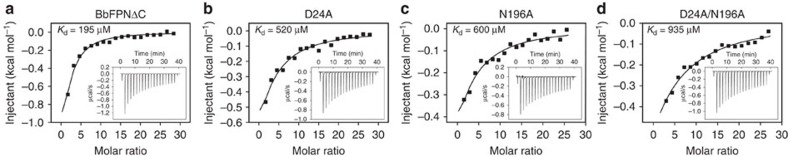
Isothermal titration calorimetry data of BbFPN. (**a**) The affinity of BbFPN toward Co^2+^ ion, measured using isothermal titration calorimetry. The data derived from (**a**) BbFPNΔC, (**b**) D24A, (**c**) N196A and (**d**) D24A/N196A double mutant are shown. The measurements were repeated twice, and similar results were obtained.

To identify the substrate-binding site, we soaked a range of transition-metal ions into the BbFPN crystals. In the data set collected from the Fe^2+^-soaked BbFPN crystal, an anomalous difference density peak was observed at the centre of the N lobe between TM1 and TM6, suggesting Fe^2+^ binding at this site (Fig. 3a,c). The side chains of Thr20, Asp24, Asn196, Ser199 and the backbone carbonyl oxygen of Thr20 coordinate the central cation, with the benzene ring of Phe200 forming a cation–π interaction (Fig. 3c). Except for Phe200, the residues involved in the cation coordination are highly conserved among the FPNs (Supplementary Fig. 1), and the corresponding residue of Asp24 in hFPN (Asp39) was previously reported to be important for both substrate binding and transport^21^. Thus, we hypothesized that the substrate transition-metal ions bind at this site. Accordingly, the D24A BbFPNΔC mutant exhibited reduced transport activity (Fig. 3d,e), consistent with the results reported for the hFPN D39A mutant^21^. We also measured the binding affinity of Co^2+^ to BbFPN, using isothermal titration calorimetry. While BbFPNΔC exhibited a 
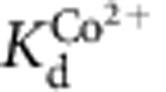
 of 195 μM, the D24A and N196A mutants showed 
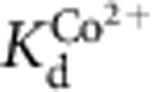
 values of 520 μM and 600 μM, respectively (Fig. 4a–c). In addition to these two single mutants, we generated the D24A/N196A double mutant, which exhibited further reduced affinity, with a 
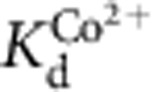
 of 935 μM (Fig. 4d). Considering the results from these structural and functional analyses, we concluded that this site is the substrate cation-binding site. The substrate-binding sites of the canonical MFS transporters are generally located at the interface between the N and C lobes^30^, as in the nitrate transporter NarU^33^ and the phosphate/proton symporter PiPT^34^. In contrast, the substrate-binding site of BbFPN lies deep inside the N lobe (Fig. 3a,b), which is a unique feature of BbFPN as compared with other MFS transporters.

A strong electron density peak was also observed at this substrate-binding site in the absence of metal soaking, suggesting that a cation from the crystallization conditions is bound to this site (Supplementary Fig. 5a,c). On the basis of the high concentration of potassium salts and the absence of any divalent cations in the crystallization conditions, we assigned K^+^ to this density peak. The distances between the K^+^ ion and the coordinating ligand atoms are 2.6–3.0 Å (Supplementary Figs 5a–d and 6a,b), consistent with the reported mean coordination distance of K^+^ ions^35^. The addition of K^+^ to the *in vitro* assay system reduced the rates of BbFPN-mediated Fe^2+^ and Co^2+^ transport, suggesting that K^+^ is bound by mimicking the cognate substrates (Supplementary Fig. 6c,d). Given that the ionic radius of K^+^ is almost twice as large as those of the BbFPN substrates (that is, 1.38 Å versus 0.69–0.83 Å; ref. 36), it seems likely that K^+^ binds to the substrate-binding site and inhibits the transport activity, rather than being competitively transported.

### Intracellular gate and its structural changes

**Figure 5:**
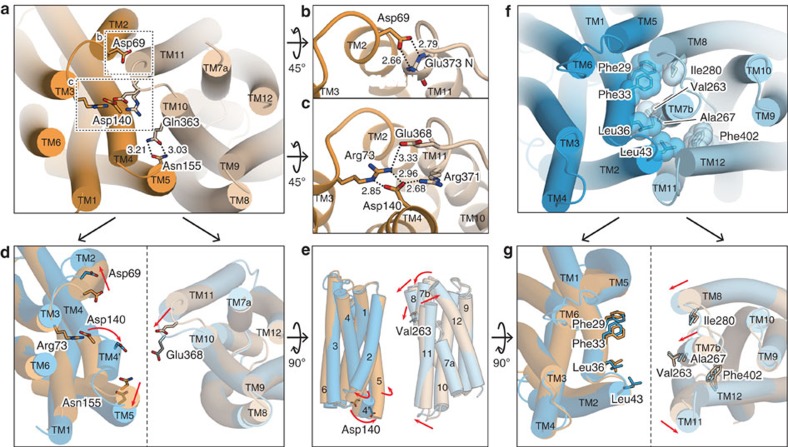
Structures of the intra- and extracellular gates and structural comparison. (**a**) Overall structure of the intracellular gate of the outward-facing state viewed from the intracellular side. The residues constituting the gate interactions are shown as stick models. (**b**,**c**) Close-up views of the intracellular gate interactions around (**b**) Asp69 and (**c**) Asp140. (**d**) Structural comparison of the intracellular side. The N lobe (left) and the C lobe (right) are separately superimposed, and viewed from the intracellular side. The disordered part of the Glu368 side chain is indicated as grey sticks. (**e**) Superimposed N lobe (left) and C lobe (right), viewed from the membrane plane. (**f**) Overall structures of the extracellular gate of the inward-facing state viewed from the extracellular side. The residues constituting the gate interactions are shown as stick models, with CPK models superimposed. (**g**) Structural comparison of the extracellular side. The N lobe (left) and the C lobe (right) are separately superimposed, and viewed from the extracellular side. In **d**,**e**,**g** the relative motions of the helices in the inward-facing state, as compared with the outward-facing state, are indicated by red arrows. All of the models are coloured in the same manner as in Fig. 2.

On the intracellular side of the outward-facing state, TM10 and TM11 of the C lobe tightly interact with TM2, TM4 and TM5 of the N lobe (Fig. 5a). Asp140 on TM4 forms salt bridges with the guanidinium groups of Arg73 on TM3 and Arg371 on TM11. Arg73 also forms a salt bridge with Glu368 on the loop between TM10 and TM11, thereby tightening the interaction network (Fig. 5a,c). Moreover, Asp69 on TM2 and Asn155 on TM5 form hydrogen bonds with the main chain nitrogen atoms of TM11 and the side chain of Gln363 on TM10, respectively (Fig. 5a,b). These interactions constitute the intracellular gate, which separates the central cavity from the intracellular side. The above-mentioned residues involved in this interaction network are all conserved in FPNs (Supplementary Fig. 1). Notably, the corresponding residues of Arg73, Asp140, Asn155 and Arg371 in hFPN (Arg88, Asp157, Asn174 and Arg489, respectively) are related to hereditary iron disorders^7^, suggesting the functional importance of the intracellular gate in the hFPN transport activity.

To visualize the structural changes around the intracellular gate during the transport cycle, we separately superimposed the N- and C-lobe structures of the inward-facing state onto those of the outward-facing state. The superimposition revealed that the intracellular side of the N lobe undergoes a relatively large structural change, including the shifts of the TM helices (Fig. 5d,e, Supplementary Movie 1). The most striking change was observed at the intracellular end of TM4. In the outward-facing state, residues 110–145 constitute a single straight TM4 helix, while in the inward-facing state, TM4 is unwound and twisted at Gly138, thus forming the short helix TM4′ (residues 139–145; Fig. 5d, Supplementary Fig. 4c,d). Consequently, Asp140 on TM4′, which interacts with Arg73 in the outward-facing state, rotates away from the Arg73 side in the inward-facing state. In addition to this unwinding of TM4, both TM2 and TM5 also undergo structural changes. The intracellular ends of these TM helices are shifted to the periphery of the transporter in the inward-facing state, which moves the Asp69 and Asn155 side chains away from the inter-lobe interface (Fig. 5d,e). In contrast to the N lobe, we did not observe any large structural changes on the intracellular side of the C lobe, except for TM11. The intracellular end of TM11 is shifted towards the centre of the transporter, thereby moving Glu368 closer to the inter-lobe interface in the inward-facing state. Taken together, these structural differences suggested that the intracellular side of the N lobe is flexible, while that of the C lobe is rather rigid. On the intracellular side of BbFPN, the induced fit of the flexible N lobe to the rigid C lobe facilitates the formation of the intracellular gate during the conformational transition from the inward- to the outward-facing states.

### Extracellular gate and its structural changes

In the inward-facing state, Phe29, Phe33, Leu36 and Leu43 in the N lobe form hydrophobic interactions with Val263, Ala267, Ile280 and Phe402 in the C lobe on the extracellular side (Fig. 5f). These hydrophobic interactions constitute the extracellular gate, and thereby separate the central cavity from the extracellular side. Among these hydrophobic residues in the extracellular gate, Phe29, Leu36 and Leu43 in the N lobe and Ile280 and Phe402 in the C lobe are highly conserved among the FPNs (Supplementary Fig. 1), indicating their importance in extracellular gate formation. It is noteworthy that the interactions in this extracellular gate are mainly hydrophobic, and no salt bridges or hydrogen bond networks are involved, in contrast to the interactions in the intracellular gate in the outward-facing state.

The comparison of the inward- and outward-facing structures further revealed the structural changes in the extracellular gate during the transport cycle: the extracellular side of the C lobe undergoes a significant conformational rearrangement involving the movements of TM7b, TM8 and TM11 (Fig. 5e,g; Supplementary Movie 1). In the inward-facing state, the entire TM7b is shifted towards the intracellular side, and its extracellular end is tilted towards the inter-lobe interface. TM8 is bent at Gly285 in the middle of the TM span, and the extracellular half of TM8 is slightly tilted towards the inter-lobe interface in the inward-facing state. In contrast to TM7b and TM8, the extracellular end of TM11 is shifted away from the inter-lobe interface, thereby avoiding steric clashes with TM2. These movements of the TM helices of the C lobe bring the hydrophobic residues closer to the inter-lobe interface, which facilitates the formation of the extracellular gate in the inward-facing state. In contrast to the C lobe, the structure of the extracellular side of the N lobe remains mostly unchanged, including the positions of the gate-forming residues (Fig. 5e,g). Taken together, the present crystal structures of BbFPN suggest that the extracellular side of the N lobe is rigid, whereas that of the C lobe is flexible. The induced fit of the flexible C lobe to the rigid N lobe forms the tight hydrophobic interaction on the extracellular side of the inward-facing structure.

### Possible mechanism of the conformational transition

**Figure 6:**
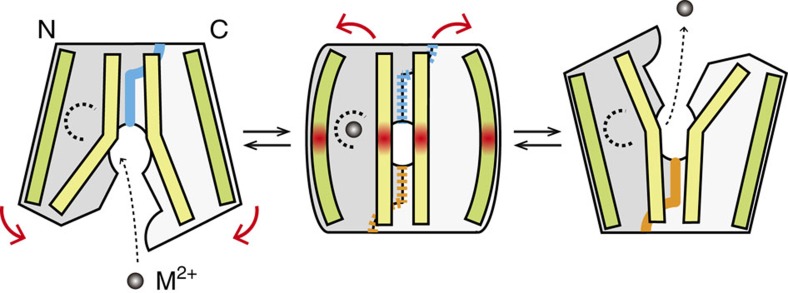
Schematic model of the transport cycle. Schematic representation of the BbFPN transport cycle. The scaffold helices are coloured green, and the helices at the inter-lobe interface are yellow. The metal-binding site is indicated as a dashed half circle. The intracellular gate interactions and the extracellular gate interactions between the two lobes are schematically represented as orange and blue lines, respectively. In the occluded state, which is shown in the middle, the distortions of the helices are indicated as red gradations. The incomplete formation of the gate interactions is indicated by dashed lines.

The present structures of BbFPN capture two extreme states of the MFS transport cycle, the outward- and inward-facing states. In addition to these states, MFS transporters are thought to form the occluded state^30^, in which the substrate-binding site is sequestered from both sides of the membrane. In the case of BbFPN, some of the interface TM helices (that is, TM1, TM2, TM5, TM7 and TM8) are bent in both the outward- and inward-facing states (Supplementary Fig. 4a–h). A structural comparison indicated that the degree of bending in these TMs slightly differs between the two states (Fig. 5d,e,g), reflecting the potential flexibility in the bent regions. Thus, we expect that the straightening of the interface TM helices allows BbFPN to form the occluded state (Fig. 6), as suggested in the previous study of the nitrate transporter NarU^33^ and NarK^37^. In contrast, TM11 and the scaffold TM helices (that is, TM3, TM6, TM9 and TM12) are straight in both the outward- and inward-facing structures (Supplementary Fig. 4i,j), allowing us to speculate that these TM helices are curved in the occluded state (Fig. 6). The distortion of these TM helices will make the occluded state energetically unfavourable, leading to the disruption of the intra- or extracellular gate, accompanying the structural rearrangements of TM4′ or TM7b, respectively (Fig. 5d,g). Along with these structural changes, the substrate binding and release will occur, resulting in the BbFPN-mediated divalent cation transport (Fig. 6).

### Homology model of human FPN and insight into FPN function

The amino-acid sequence similarity and the conservation of functionally important residues between BbFPN and hFPN allowed us to construct a reliable homology model of hFPN based on the present structure. The model was built using the outward-facing structure of BbFPN as the template, as it was determined at a higher resolution than the inward-facing structure. The residues involved in the cation coordination in the BbFPN structures are well conserved (Supplementary Fig. 1), suggesting that the substrate cation is similarly coordinated by the corresponding residues (that is, Ser35, Asp39, Asn212 and Ser215) in hFPN. In the homology model of hFPN, these four residues are clustered with suitable geometry for the cation coordination, as observed in the BbFPN structures (Supplementary Fig. 7a). In contrast, Phe200 of BbFPN, which coordinates the metal ion by a cation–π interaction in the BbFPN structures, is replaced by methionine in hFPN (Supplementary Figs 1 and 7a). This difference suggests that the thioether sulfur atom of this methionine may be involved in the cation recognition. Similar cation coordination is also observed in ScaDMT^24^ (Supplementary Fig. 7b), a bacterial homologue of the human iron importer DMT1 (ref. 38). ScaDMT uses the methionine sulfur atom to specifically recognize transition-metal ions, including Mn^2+^ and Fe^2+^, at its substrate-binding site. The coordination of cations by the thioether group of methionine may be a common mechanism employed by these transition-metal transporters for the specific recognition of their substrate cations.

**Figure 7:**
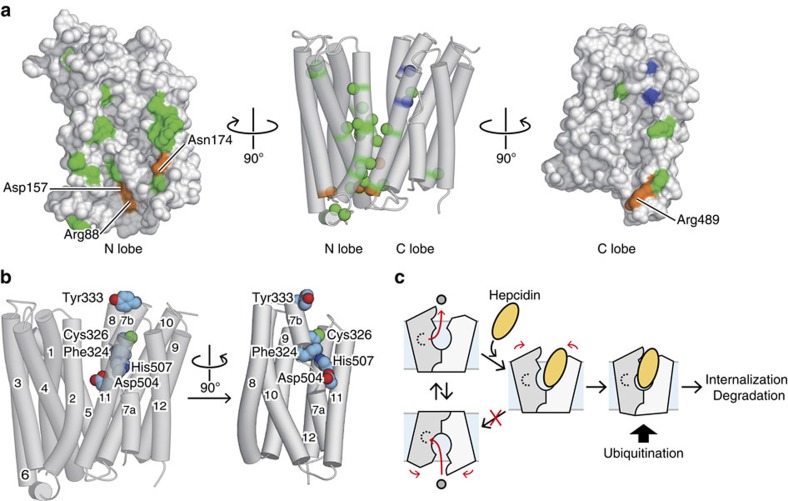
Mapping of functionally important residues on the hFPN homology model. (**a**) Mapping of disease-related mutation sites on the hFPN homology model. The residues involved in the intracellular gate interactions and the hepcidin binding are coloured orange and blue, respectively. Other mutation sites are coloured green. In the middle panel, TM helices are represented as semitransparent cylinders, and the Cα atoms of the mutation sites are indicated by CPK spheres. The surface representation of the N lobe, viewed from the inside of the central cavity, is shown in the left panel, while that of the C lobe is shown in the right panel. (**b**) Mapping of the hepcidin-binding residues on the hFPN homology model. The side chains of these residues are represented by CPK models. In the right panel, the N lobe is omitted for clarity. (**c**) Possible model of the hepcidin-mediated inhibition of hFPN. Hepcidin, represented as the yellow oval, binds to the hFPN C lobe, inhibiting its state transition toward the inward-facing state. Hepcidin binding may also change the conformation of the intracellular side of hFPN, which triggers the ubiquitination and subsequent internalization.

The hFPN homology model also provides a mechanistic interpretation for the disease-related mutations that cause hereditary hemochromatosis^7^. The mutation sites are mainly mapped onto the inter-lobe interface (Fig. 7a). Among them, Arg88, Asp157, Asn174 and Arg489, which correspond to Arg73, Asp140, Asn155 and Arg371 in BbFPN, respectively (Fig. 7a), are located on the intracellular side and form the intracellular gate interactions. Other mutation sites are clustered around these residues, and may contribute to the inter-lobe contacts. Thus, these disease-related mutations may destabilize the inter-lobe interactions, thereby affecting the stable formation of the intracellular gate and reducing the Fe^2+^ transport activity of hFPN.

Our homology model of hFPN further provides crucial insights into the hepcidin–hFPN interaction. Previous biochemical experiments revealed the direct interaction between hFPN and hepcidin^9^, in which Phe324, Cys326, Tyr333, Asp504 and His507 play important roles^22^,^39^,^40^,^41^. Among these residues, Cys326 has been extensively studied. Binding experiments using ^125^I-labelled hepcidin and the chemical modification of free thiols revealed that the thiol group of Cys326 is essential for hepcidin binding^41^. The binding experiments also indicated that the hepcidin–hFPN interaction involves disulfide bond formation between Cys326 of hFPN and Cys7 of hepcidin^39^,^41^. On the basis of these results, Cys326 is widely accepted as the hepcidin-binding site. Given the predicted topology of hFPN^18^ and the high accessibility of Cys326 from the extracellular side, the hepcidin-binding site is thought to be located on the extracellular loop of hFPN. In our hFPN homology model, however, all of the hepcidin-binding residues mentioned above are clustered inside the central cavity except for Tyr333, which is located at its entrance, with Cys326 located at the intracellular end of TM7b (Fig. 7b). Therefore, it is likely that hepcidin enters the central cavity between the N and C lobes, and thereby interacts with the hepcidin-binding site located on the C lobe (Supplementary Fig. 7c). Recent studies demonstrated that hepcidin binding to FPN induces the ubiquitination of lysine residues on the intracellular loop between TM6 and TM7, which subsequently triggers the internalization of the hepcidin–FPN complex^12^,^13^. Thus, hepcidin binding to the central cavity may affect the conformations of the intracellular loops that harbour the ubiquitination sites, thereby increasing their accessibility to the ubiquitin ligases.

Furthermore, the proposed hepcidin-binding mode suggests that its binding arrests the conformational transition of hFPN from the outward-facing state to the inward-facing state. This may prevent the disruption of the intracellular gate and thereby inhibit the access of Fe^2+^ from the cytoplasm to the substrate-binding site (Fig. 7c), leading to the reduction of hFPN-mediated iron efflux. The direct influence of hepcidin binding on the hFPN activity cannot be easily measured due to the rapid internalization, and thus has not been extensively examined. We acknowledge that our proposed model is still speculative, and further validation by biochemical analyses is necessary. Nonetheless, it is tempting to speculate that hepcidin may directly modulate the hFPN activity, thus facilitating the rapid control of the plasma iron levels.

## Methods

### Protein expression and purification

The gene encoding *B. bacteriovorus* HD100 Bd2019 (BbFPN, Genomic DNA GenBank ID: BX842651.1, Protein ID: CAE79867.1) was synthesized and cloned into the pWaldo-GFPe vector^42^, with the tobacco etch virus (TEV) protease cleavage site inserted between the C terminus of BbFPN and GFP-His_8_. For the crystallization construct, eight residues at the C terminus were deleted (BbFPNΔC). BbFPN was expressed in the *Escherichia coli* C41 (DE3) strain. Transformed cells were grown at 37 °C to an *OD*_600_ of 0.9–1.0, and then protein expression was induced with 0.2 mM isopropyl-β-D-thiogalactopyranoside at 20 °C for 18 h. For the FSEC analysis, harvested cells were resuspended in buffer A (20 mM MES, pH 6.0, 500 mM NaCl, 10% glycerol, 50 μM ZnCl_2_, 0.1 mM phenylmethylsulfonyl fluoride (PMSF)) and disrupted by sonication. Subsequently, 1% *n*-dodecyl-β-D-maltoside (DDM) was added to the sample, and mixed for 1 h to solubilize the protein. After clearing the crude lysates by ultracentrifugation (70,000*g*, 15 min), the supernatant was analysed by the fluorescent size exclusion chromatography, as previously described^23^. For large-scale purification, we used two different purification protocols, protocol A and protocol B. In protocol A, harvested cells were lysed in buffer A, and subsequently disrupted using a Microfluidizer processor (Microfluidics) by three passages at 15,000 p.s.i. After the removal of debris by centrifugation (12,500*g*, 30 min, 4 °C), the supernatant was ultracentrifuged (125,000*g*, 1 h, 4 °C) to collect the membrane fraction. The collected membrane pellet was then solubilized in buffer A containing 1% DDM, for 2 h at 4 °C. Insoluble materials were removed by ultracentrifugation (125,000*g*, 30 min, 4 °C), and the supernatant was applied to Ni-NTA superflow resin (Qiagen). After mixing the samples for 1.5 h at 4 °C, the resin was washed with buffer A containing 0.03% DDM and 20 mM imidazole, pH 6.6. The resin was further washed with similar buffer containing 0.01% lauryl maltose neopentyl glycol (LMNG) instead of DDM, to change the detergent. After these wash processes, BbFPN was eluted with 300 mM imidazole, pH 6.6. The sample was subsequently dialyzed to remove the imidazole, with His-tagged TEV protease added for GFP-His_8_ cleavage. After overnight dialysis, the sample was loaded onto Ni-NTA resin to remove the TEV protease and GFP-His_8_. The flow-through fraction containing BbFPN was collected and concentrated for subsequent gel filtration (Superdex 200 Increase 10/300 GL, GE Healthcare) in buffer A containing 0.004% LMNG, with the concentration of glycerol reduced to 5%. In purification protocol B, buffer B (20 mM Tris, pH 8.0, 500 mM NaCl, 10% glycerol, 0.1 mM PMSF) was used instead of buffer A, and accordingly, the imidazole was changed to pH 8.0. The glycerol concentration of the final gel filtration buffer was set to 10%. Other purification procedures were conducted in the same manner as in protocol A. In both protocols A and B, the purified BbFPN was concentrated to ∼10 mg ml^−1^ after gel filtration, using an Amicon Ultra filter (MWCO 100 kDa).

Purification protocol A yielded slightly more stable protein samples than protocol B, and generated larger crystals with higher reproducibility. For the functional analyses, however, the presence of Zn^2+^ in the buffer may affect the results and therefore, protocol B was employed. Interestingly, the form I crystals or similar crystals were obtained from the protein samples purified using either protocol, while the form II crystals were only obtained from the protein samples purified using protocol B.

### Crystallization

The purified BbFPN was reconstituted into the lipidic cubic phase (LCP) by mixing with monoolein at a 2:3 protein to lipid ratio (wt/wt), using the twin-syringe mixing method^27^. After the reconstitution, 40 nl LCP drops were dispensed onto either LCP lipidic cubic phase screening plates (SWISSCI) or Laminex glass plates (Molecular Dimensions), and overlaid with 800 nl of precipitant solution using the Mosquito LCP crystallization robot (TTP LabTech). Initial crystallization trials were performed using the MemMeso crystallization screen (Molecular Dimensions). For the Hg-based phase determination, both the native crystals and the Hg-derivative crystals were obtained from the protein sample purified with protocol A. These crystals were obtained at 20 °C in the precipitant solution containing 100 mM Tris, pH 7.5, 50–200 mM K-formate, 50–100 mM (NH_4_)_2_HPO_4_, and 26–32% PEG550MME. For the preparation of the Hg-derivative crystals, the precipitant solution supplemented with 1 mM CH_3_HgCl was injected into the well containing the native crystals and incubated for 2.5 h before harvesting them. The Fe^2+^-soaked crystal was also prepared in a similar manner, using 5 mM FeSO_4_ instead of CH_3_HgCl, and was incubated for 12 h. All the crystals mentioned above are similar to the form I crystals in the lattice parameters. To obtain the form I and form II crystals, protein samples purified with protocol B were used. LCP reconstitution and crystallization were conducted as described above, and the crystals were incubated at 20 °C. The form I crystals were grown in the precipitant solution containing 100 mM Tris, pH 8.5, 100 mM NaK-tartrate, 100 mM KNO_3_, and 30% PEG550MME, and the form II crystals were grown in the precipitant solution containing 100 mM Na-acetate, pH 4.0, 200–300 mM K-formate, 10 mM ZnSO_4_, 10–20 mM ZnCl_2_, and 27–30% PEG550MME. All of the crystals were directly harvested from the crystallization plates and flash frozen in liquid nitrogen.

### Data collection and structure determination

All diffraction data sets were collected at SPring-8 BL32XU, using a micro-focused X-ray beam^43^, and processed using the programme XDS^44^. For the phase determination, the data sets collected from the native crystal and the Hg-derivative crystal were analysed using the programs SHELXC and D (ref. 45), and one Hg atom site was identified. Based on this information, the initial phases were calculated using the programme SHARP^46^, by the single isomorphous replacement with anomalous scattering method. The initial model was built using the programme PHENIX AutoBuild^47^. The model was further built manually using COOT^48^ and refined using PHENIX^49^, and finally the structure was determined at 2.5 Å resolution. By utilizing this structure as the search model, the form I structure was determined by molecular replacement with the programme PHASER^50^. The form II structure was determined by molecular replacement using the refined form I structure, with separate searches for the N lobe and the C lobe. For the model building and the refinement of both the form I and form II structures, COOT^48^ and PHENIX^49^ were used. The data collection and refinement statistics are summarized in Table 1. All molecular graphics were prepared using CueMol (http://www.cuemol.org/).

### Preparation of proteoliposomes

*E. coli* polar lipid extract (Avanti Polar Lipids) was dried, and subsequently resuspended and solubilized in buffer containing 25 mM HEPES, pH 7.0, 100 mM NaCl and 0.6% Na-cholate, at a 12.5 mg ml^−1^ concentration. The protein purified with protocol B was added to this solubilized lipid at a 1:40 protein to lipid ratio (wt/wt). Bio-Beads SM-2 (Bio-Rad) were immediately added after mixing the protein and the lipid, and the sample was further mixed at 4 °C for 24 h, to remove the detergent. After the removal of the Bio-Beads, the sample was sonicated for 30 s, using a Bioruptor (CosmoBio), and subsequently flash frozen in liquid nitrogen and stored at −80 °C. To monitor the pH dependence of the transport, proteoliposomes at different pH values were prepared in a similar manner, using different buffers (MES pH 6.0, MES pH 6.5, HEPES pH 7.5 or HEPES pH 8.0) instead of HEPES pH 7.0. Control liposomes without protein were prepared using the same procedure. Due to the relatively higher stability of the protein samples, BbFPNΔC constructs were used for the reconstitution, unless otherwise stated.

### Metal uptake assay using calcein

For the metal uptake assays, 10 μl of proteoliposomes or control liposomes were mixed with 10 μl of assay buffer containing 25 mM HEPES, pH 7.0, 100 mM NaCl, and 500 μM calcein, and subsequently subjected to three freeze-thaw cycles. The sample was then sonicated for 30 s, using a Bioruptor (CosmoBio). To remove the excess calcein remaining in the outer solution, the sample was loaded onto Sephadex G50 fine (GE Healthcare) gel filtration medium. After diluting the collected liposomes with 490 μl of assay buffer, the sample fluorescence was monitored using an F7000 fluorescence spectrophotometer (Hitachi), with *λ*_ex_=494 nm and *λ*_em_=516 nm. At 500 s after starting the measurement, the metal cations (100 μM; 50 μM for Fe^2+^) were added to the outer solution of the liposomes, and the change in the calcein fluorescence was recorded for the next 20 min (5 min for Fe^2+^). For the Fe^2+^-influx measurement, the metal cation solution (1:1 molar ratio of ammonium iron sulfate and sodium dithionite diluted in the assay buffer) was prepared immediately before the addition to the outer solution, to avoid the oxidation of Fe^2+^. All measurements were performed at 37 °C. In the Fe^2+^, Co^2+^, Mn^2+^ and Ni^2+^ uptake assays shown in Fig. 1a–d, the ionophore calcimycin (Sigma) was added at a concentration of 1 μM at the end of the measurement to fully quench the calcein fluorescence. For the decoupling experiment, 1 μM of gramicidin (Sigma) was added to the outer solution before starting the measurement. To create the Na^+^ concentration gradient, the NaCl concentration in the outer solution was changed to 50, 75, 125 or 150 mM, and choline chloride was used to balance the osmolarity. To change the pH of the outer solution, different buffers (MES pH 6.0, MES pH 6.5, HEPES pH 7.5 or HEPES pH 8.0) were used instead of HEPES pH 7.0. To measure the K^+^-mediated inhibition of the transport, 200 mM KCl was added to both the inside and outside of the liposomes. All measurements were repeated at least three times, and similar results were obtained.

### Isothermal titration calorimetry

Protein samples were purified with protocol B, and concentrated to 0.05–0.06 mM (2.5–2.9 mg ml^−1^). The samples were subsequently dialyzed overnight against buffer containing 20 mM Tris, pH 8.0, 500 mM NaCl, 10% glycerol and 0.004% LMNG. The solution used for titration was prepared by adding CoCl_2_ to the dialysis buffer, at a concentration of 8–10 mM. The ITC measurements were performed with a MicroCal iTC 200 calorimeter (GE Healthcare) at 20 °C. The Co^2+^ was injected 20 times (0.4 μl for injections 1 and 20, and 2 μl for injections 2–19), with 120 s intervals between injections. The background data obtained from the buffer sample were subtracted before the data analysis. The data were fitted using the Origin7 software package (MicroCal). Measurements were repeated twice, and similar results were obtained. The BbFPNΔC constructs were used for the measurements.

### Homology modelling of hFPN

Based on the outward-facing structure of BbFPN, homology models of hFPN were generated using the programme MODELLER^51^. The orientations of the residues around the metal-binding site were manually adjusted, using the programme COOT^48^. The Fe^2+^ ion was placed at the position corresponding to the K^+^ in the BbFPN structure. The hepcidin–hFPN docking model was constructed manually, using COOT^48^.

## Additional information

**How to cite this article:** Taniguchi, R. *et al*. Outward- and inward-facing structures of a putative bacterial transition-metal transporter with homology to ferroportin. *Nat. Commun.* 6:8545 doi: 10.1038/ncomms9545 (2015).

## Supplementary Material

Supplementary Information Supplementary Figures 1-7 and Supplementary References

Supplementary Movie 1 Intra-lobe conformational changes of BbFPN between the outward- and inward-facing states

## Figures and Tables

**Table 1:** Data collection and refinement statistics.

	Native	Hg derivative*	Fe^2+^ soaked*	Form I (outward)	Form II (inward)*
*Data collection*					
Wavelength (Å)	1.0000	1.0000	1.4500	1.0000	1.2820
Space group	*P*2_1_2_1_2_1_	*P*2_1_2_1_2_1_	*P*2_1_2_1_2_1_	*P*2_1_2_1_2_1_	*P*2_1_2_1_2_1_
Cell dimensions					
*a*, *b*, *c* (Å)	57.15, 86.19, 97.07	57.40, 86.14, 96.28	57.08, 86.17, 96.71	56.64, 84.63, 96.81	63.53 70.68 101.79
*α, β, γ* (°)	90, 90, 90	90, 90, 90	90, 90, 90	90, 90, 90	90, 90, 90
Resolution (Å)	43.1–2.41 (2.49–2.41)^†^	47.6–2.50 (2.59–2.50)	49.2–3.00 (3.11–3.00)	48.4–2.20 (2.28–2.20)	42.9–3.30 (3.42–3.30)
*R*_merge_ (%)	12.6 (80.1)	19.1 (99.0)	23.4 (86.0)	15.5 (111.0)	23.1 (150.6)
*I*/σ*I*	11.2 (2.1)	10.2 (2.3)	10.4 (2.7)	10.6 (1.8)	7.8 (2.0)
Completeness (%)	99.5 (95.4)	99.5 (95.9)	100.0 (100.0)	99.9 (99.6)	98.5 (99.3)
Redundancy	5.9 (5.6)	7.8 (7.7)	7.6 (7.5)	7.3 (7.2)	4.0 (3.9)
CC_1/2_ (%)	99.7 (71.5)	99.6 (73.7)	99.6 (78.6)	99.7 (64.1)	98.7 (46.0)
					
*Refinement*					
Resolution (Å)	2.50		3.00	2.20	3.30
No. reflections	17154		18454	24182	13103
*R*_work/_ *R*_free_ (%)	22.1/25.4		22.4/26.9	19.7/24.0	23.5/28.5
No. of atoms					
Protein	3016		3014	3088	2980
Ligand/ion	0		1	157	16
Water	17		0	58	6
B-factors					
Protein	40.9		26.7	32.4	59.6
Ligand/ion	—		26.0	50.9	61.2
Water	34.5		—	33.2	46.1
R.m.s.d.					
Bond lengths (Å)	0.004		0.002	0.007	0.002
Bond angles (°)	0.770		0.604	1.033	0.642
Ramachandran plot					
Favoured (%)	98.72		98.72	98.25	98.01
Allowed (%)	1.28		1.28	1.75	1.99
Outliers (%)	0.00		0.00	0.00	0.00

^*^Friedel pairs are treated separately. ^†^Highest resolution shell is shown in parentheses.
