# Supplementary material for: Outward- and inward-facing structures of a putative bacterial transition-metal transporter with homology to ferroportin
Source: Nat Commun. 2015 Oct 13;6:8545. doi: 10.1038/ncomms9545 (PMC4633820; doi:10.1038/ncomms9545)
Supplement: Supplementary Information — Supplementary Figures 1-7 and Supplementary References [file ncomms9545-s1.pdf]

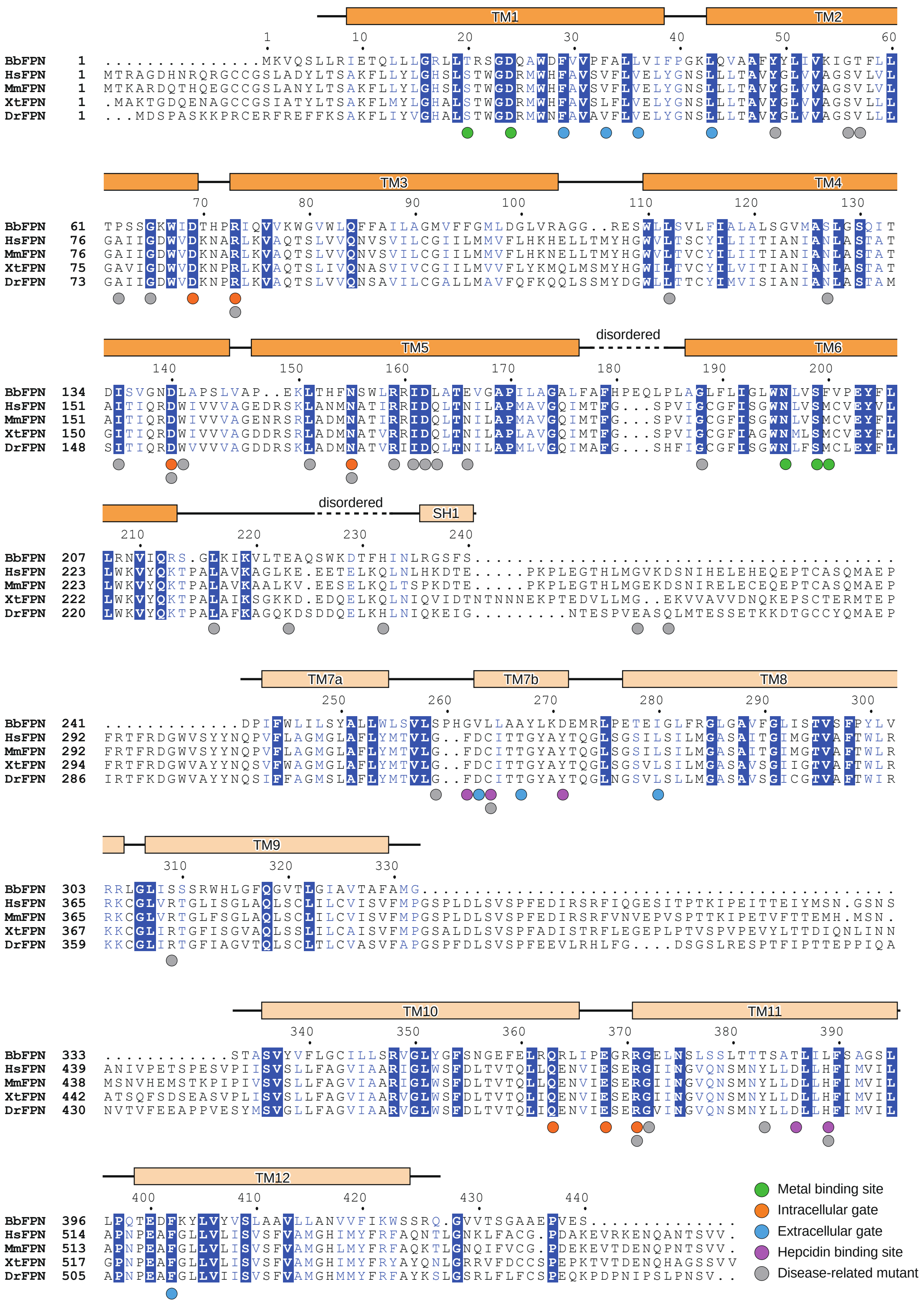

TM1
TM2
TM3
TM4
TM5
TM6
disordered
SH1
TM7a
TM7b
TM8
TM9
TM10
TM11
TM12
BbFPN
HsFPN
MmFPN
XtFPN
DrFPN
Metal binding site
Intracellular gate
Extracellular gate
Hepcidin binding site
Disease-related mutant

Supplementary Figure 1. Sequence alignment of BbFPN with eukaryotic FPNs.

The amino acid sequence alignment of BbFPN, *Homo sapiens* FPN (HsFPN), *Mus musculus* FPN (MmFPN), *Xenopus tropicalis* FPN (XtFPN) and *Danio rerio* FPN (DrFPN). Strictly conserved residues are highlighted in blue boxes, and highly conserved residues are indicated as blue letters. The secondary structure of the BbFPN outward-facing state is indicated above the alignment. Below the alignment, the residues involved in the metal coordination, the intracellular gate interactions, and the extracellular gate interactions in the BbFPN structures are highlighted with green, orange and blue circles, respectively. The hepcidin-binding sites of hFPN are highlighted with magenta circles. The positions of the disease-related mutations of hFPN[1] are indicated with gray circles.

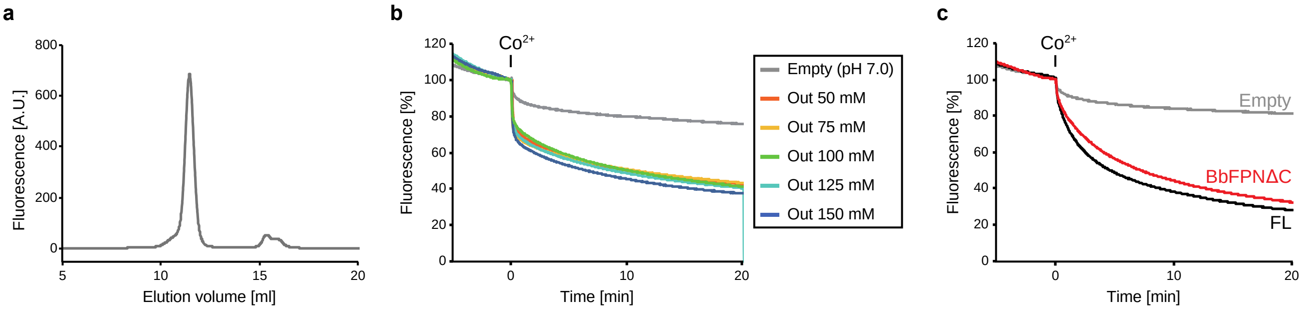

Supplementary Figure 2. Characterization of the BbFPN protein.

(**a**) The fluorescent size exclusion chromatography[2] trace of full-length BbFPN fused with GFP. The void volume is approximately 8 ml. The main peak represents the solubilized BbFPN-GFP. (**b**) The transport activity of BbFPN measured with Na^{+} concentration gradients across the membrane. Time-dependent quenching of calcein fluorescence inside the liposomes is shown. The Na^{+} concentration of the inner solution was set to 100 mM, while those of the outer solution were 50, 75, 100, 125, and 150 mM. All measurements were conducted at pH 7.0. (**c**) Comparison of the Co^{2+} transport activities between full-length BbFPN and BbFPNΔC, measured using the fluorescent indicator calcein. The gray trace indicates the control liposome data. The black and red traces indicate the data derived from the full-length BbFPN and BbFPNΔC, respectively. The liposome measurements were repeated three times, and representative data are shown.

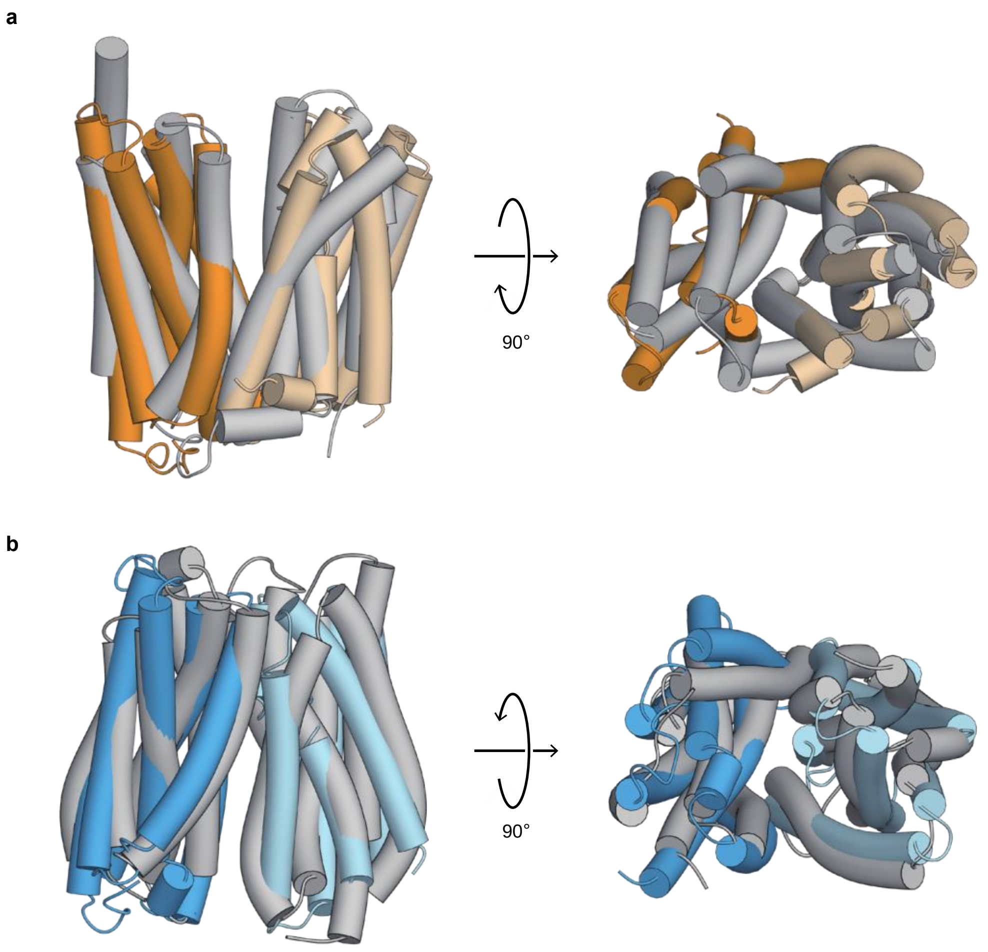

Supplementary Figure 3. Superimposition on MFS transporter structures.

(**a**) The outward-facing structure of BbFPN superimposed on the outward open structure of FucP[3] (PDB ID: 3O7Q). The BbFPN structure is colored in the same manner as in Fig. 2a. The FucP structure is colored gray, and the residues between 180-192 are omitted for clarity. The left panel is viewed from the membrane plane, and the right panel is viewed from the extracellular side. (**b**) The inward-facing structure of BbFPN superimposed on the inward-open structure of GkPOT[4] (PDB ID: 4IKV). The BbFPN structure is colored in the same manner as in Fig. 2b. The structure of GkPOT is colored gray, with the N-terminal loop (residues 2-19) and two additional helices (residues 221-282) omitted for clarity. The left panel is viewed from the membrane plane, and the right panel is viewed from the intracellular side.

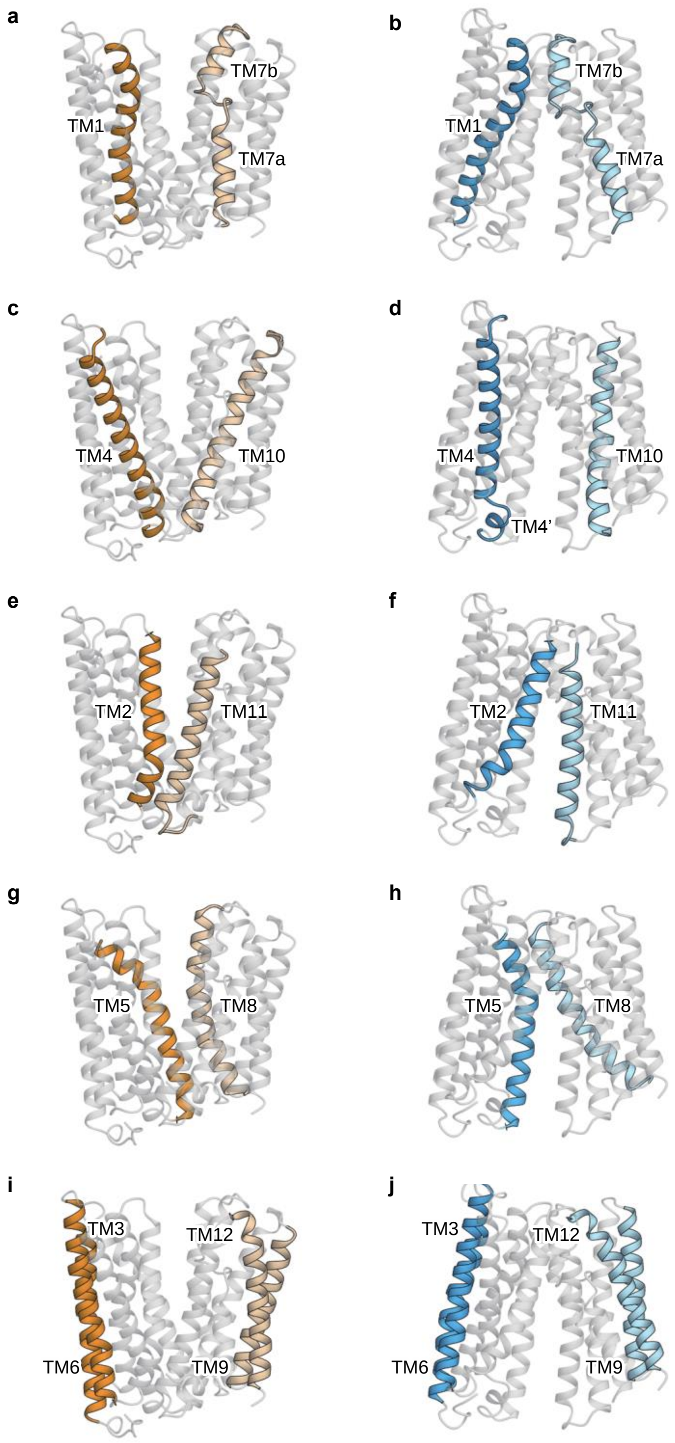

Supplementary Figure 4. Transmembrane helices in BbFPN structures.

The symmetry-related transmembrane helices in (**a, c, e, g, i**) the outward-facing structure and (**b, d, f, h, j**) the inward-facing structure of BbFPN. (**a, b**) TM1 and TM7, (**c, d**) TM4 and TM10, (**e, f**) TM2 and TM11, (**g, h**) TM5 and TM8, and (**i, j**) the scaffold helices (TM3, TM6, TM9, TM12) are separately highlighted in each panel. All of the models are viewed from the same orientation as in Fig. 2, and the highlighted helices are colored in the same manner. The background helices are colored gray with semitransparent representation.

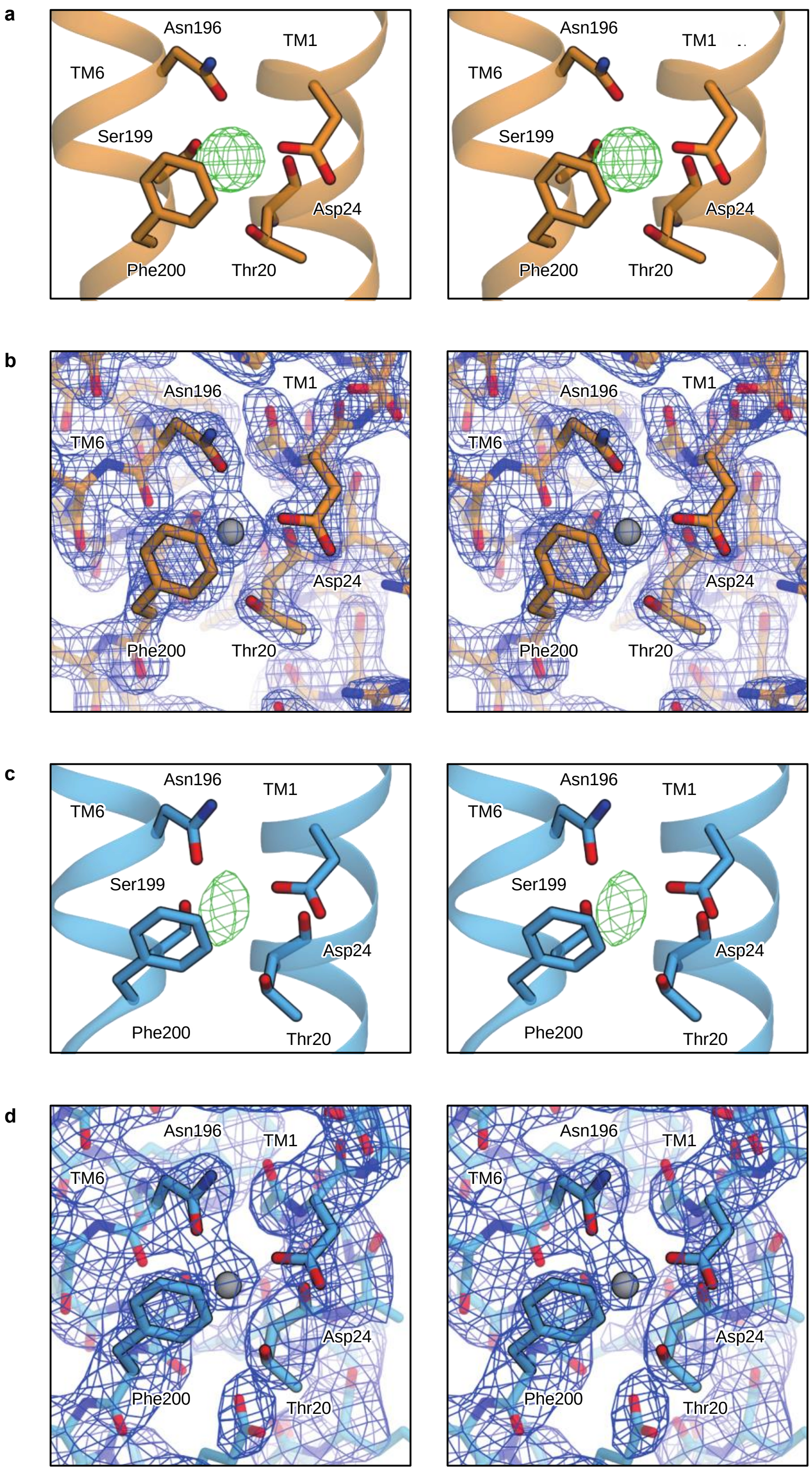
a
Asn196
TM1
TM6
Ser199
Asp24
Phe200
Thr20
b
Asn196
TM1
TM6
Asp24
Phe200
Thr20
c
Asn196
TM1
TM6
Ser199
Asp24
Phe200
Thr20
d
Asn196
TM1
TM6
Asp24
Phe200
Thr20

Supplementary Figure 5. Electron density maps of the substrate-binding site.

Stereo views of the electron density maps of the BbFPN substrate-binding site in (**a, b**) the outward-facing and (**c, d**) the inward-facing structures. (**a**) The *F*o – *F*c omit map calculated without bound K^+ (contoured at 6 σ) is shown as green mesh. The residues coordinating the K^+ ion are shown as stick models. (**b**) The 2*F*o – *F*c electron density map (contoured at 1.5 σ) is shown as blue mesh, with the bound K^+ represented as a gray sphere. (**c**) The *F*o – *F*c omit map calculated without bound K^+ (contoured at 5 σ) is shown as green mesh. The residues coordinating the K^+ ion are shown as stick models. (**d**) The 2*F*o – *F*c electron density map (contoured at 1.2 σ) is shown as blue mesh, with the bound K^+ represented as a gray sphere. The residues coordinating the K^+ ion are shown as stick models. The outward-facing structure models are colored orange, while the inward-facing structure models are colored blue. Panels **a** and **b** are viewed from the same orientation as in Fig. 2a. The orientations of panels **c** and **d** are adjusted to those of panels **a** and **b** for the comparison. In panels **b** and **d**, Ser199 is not labeled for clarity.

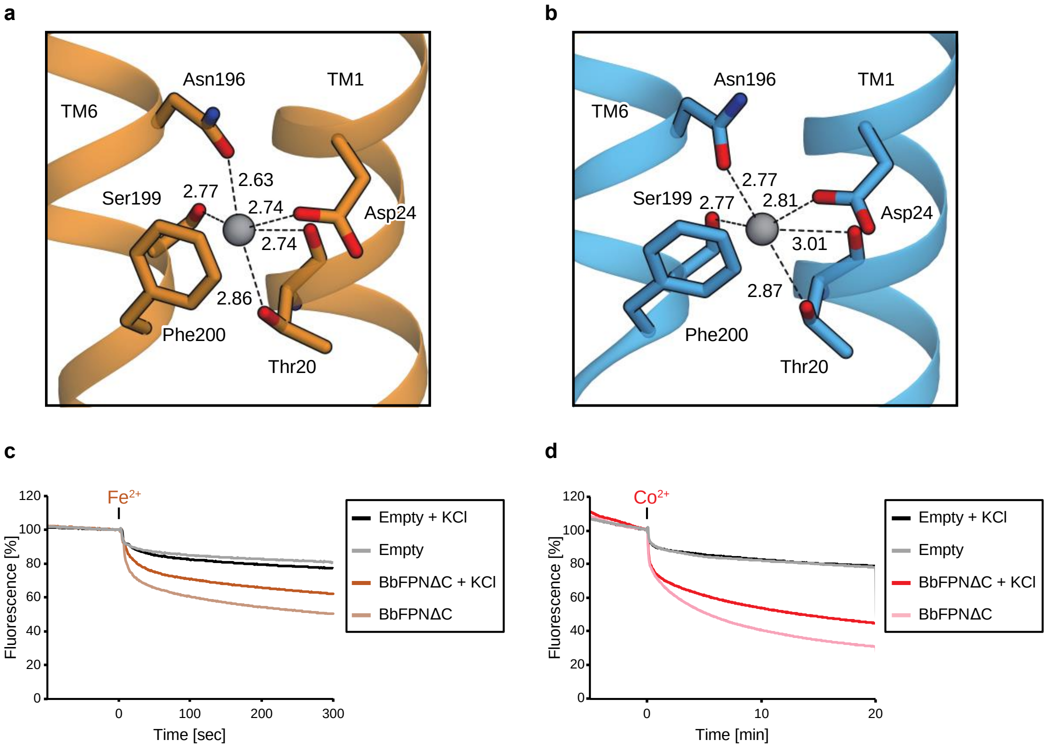

Supplementary Figure 6. K^+ ion binding to the BbFPN substrate-binding site.

(**a**) Close-up view of the K^+ coordination in the outward-facing structure of BbFPN. The ion-protein interactions are represented by dashed lines, with the interaction distances (Å) shown nearby. The K^+ ion is depicted by a gray sphere. (**b**) The same region in the inward-facing structure, shown in the same manner as in **a**. (**c, d**) The K^+-mediated inhibition of the (**c**) Fe^{2+} and (**d**) Co^{2+} transport activities of BbFPN. Time-dependent quenching of calcein fluorescence is shown. The data derived from proteoliposomes under K^+-free conditions are represented by (**c**) pale brown and (**d**) pink traces, while the data measured in the presence of 200 mM KCl are shown as (**c**) brown and (**d**) red traces. The data derived from control liposomes with and without KCl are indicated as black and gray traces, respectively. The liposome measurements were repeated three times, and representative data are shown.

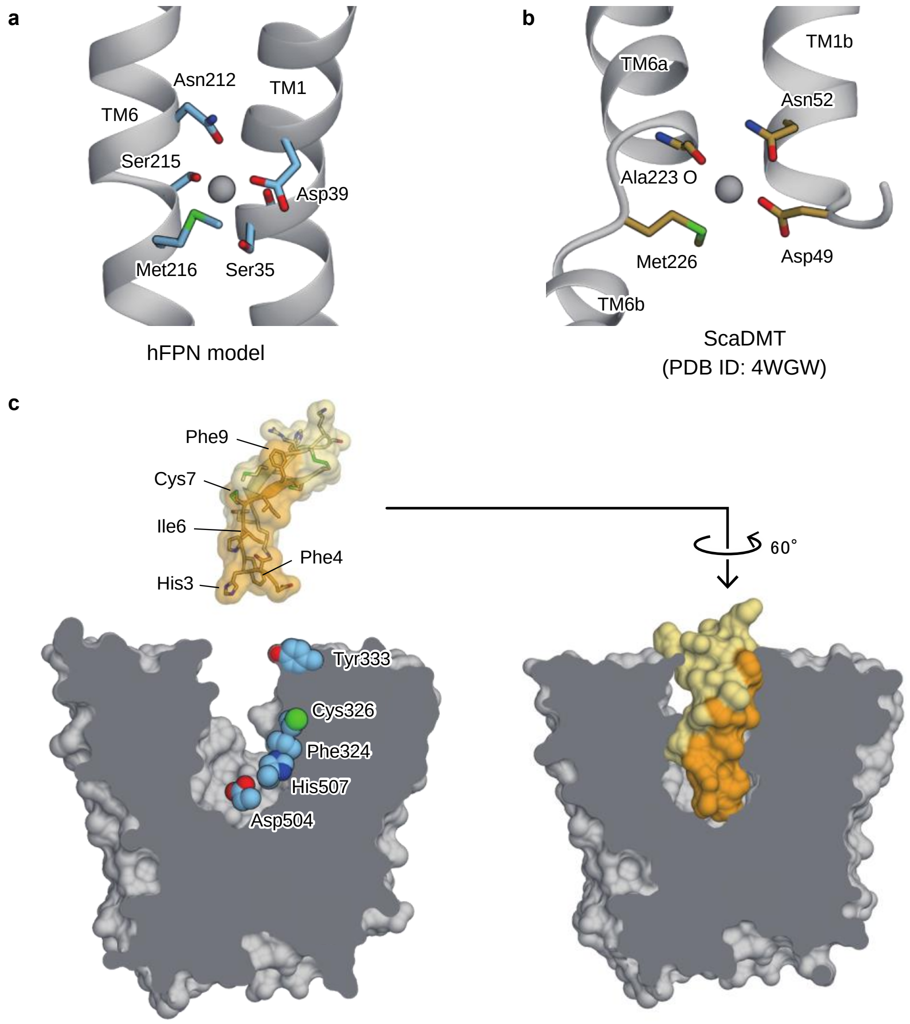

Supplementary Figure 7. hFPN homology model.

(**a**) The putative substrate-binding site in the hFPN homology model. The residues corresponding to Thr20, Asp24, Asn196, Ser199 and Phe200 in BbFPN are shown as stick models. The Fe^{2+} ion is modeled at the center of this site, and is depicted by a gray sphere. (**b**) The substrate-binding site of the ScaDMT structure[5] (PDB ID: 4WGW), with the bound Mn^{2+} depicted by a gray sphere. (**c**) The hFPN homology model and the hepcidin structure determined by NMR spectroscopy[6] (PDB ID: 2KEF), shown on the same scale. The N-terminal nine residues of hepcidin, which are important for the hepcidin-hFPN interaction[7], are highlighted in yellow, and particularly important residues for the interaction are labeled. The hFPN homology model is shown with the cross section of the surface representation. In the left panel, the hepcidin-binding residues of hFPN are depicted as CPK models. In the right panel, a docking-model of hFPN and hepcidin is shown. The hFPN homology model is viewed from the same orientation as in the left panel of Fig. 7b.

Supplementary References

1. Détivaud, L. *et al.* Ferroportin diseases: functional studies, a link between genetic and clinical phenotype. *Hum. Mutat.* **34,** 1529–36 (2013).

2. Kawate, T. & Gouaux, E. Fluorescence-detection size-exclusion chromatography for precrystallization screening of integral membrane proteins. *Structure* **14,** 673–81 (2006).

3. Dang, S. *et al.* Structure of a fucose transporter in an outward-open conformation. *Nature* **467,** 734–8 (2010).

4. Doki, S. *et al.* Structural basis for dynamic mechanism of proton-coupled symport by the peptide transporter POT. *Proc. Natl. Acad. Sci. U. S. A.* **110,** 11343–8 (2013).

5. Ehrnstorfer, I. A., Geertsma, E. R., Pardon, E., Steyaert, J. & Dutzler, R. Crystal structure of a SLC11 (NRAMP) transporter reveals the basis for transition-metal ion transport. *Nat. Struct. Mol. Biol.* **21,** 990–6 (2014).

6. Jordan, J. B. *et al.* Hepcidin revisited, disulfide connectivity, dynamics, and structure. *J. Biol. Chem.* **284,** 24155–67 (2009).

7. Preza, G. C. *et al.* Minihepcidins are rationally designed small peptides that mimic hepcidin activity in mice and may be useful for the treatment of iron overload. *J. Clin. Invest.* **121,** 4880–4888 (2011).
